# Moist verrucous plaques of the nail matrix and nail bed: A case of probable ungual pemphigoid vegetans

**DOI:** 10.1016/j.jdcr.2025.10.005

**Published:** 2025-10-11

**Authors:** Xue Ling Ang, Yihan Li, Chia Chun Ang

**Affiliations:** a Department of Dermatology, Singapore General Hospital, Singapore; b Department of Anatomical Pathology, Singapore General Hospital, Singapore

**Keywords:** bullous pemphigoid, nail, pemphigoid vegetans

## Introduction

Autoimmune blistering diseases can affect the nail unit because of the expression of common desmosome and hemidesmosome target antigens that it shares with the skin.^1^ We present a patient with a rare presentation of isolated nail unit pemphigoid vegetans.

## Case report

A 60-year-old Malay man with no significant medical history presented with moist, malodorous, verrucous plaques replacing the nail bed and matrix of both halluces. This started 5 months earlier, when he noticed a crack on the left big toenail after a knock injury with subsequent serous discharge from the affected toe and acute paronychia of the proximal and lateral nailfolds. The right big toe soon became similarly affected. His condition did not improve despite empirical treatment with a 2-month course of oral itraconazole, a 1-week course of oral amoxicillin-clavulanic acid, topical fusidic acid-betamethasone valerate cream, and miconazole cream given by his primary care physician before our consultation.

**Fig 1:**
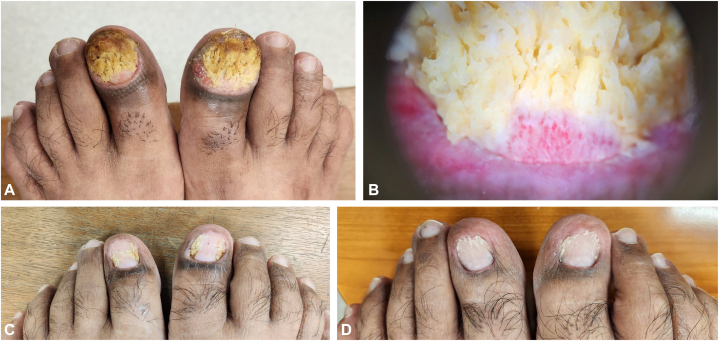
Ungual pemphigoid vegetans. Initial acute paronychia of bilateral halluces (not shown) followed by moist verrucous plaques replacing the nail matrix and nail bed of bilateral halluces (**A,** July 2024) and the close-up image of the proximal nailfold, distal nail matrix, and nail bed on dermatoscopy (**B,** August 2024. Image taken using Dermlite DL200 Hybrid at ×10 magnification). There was a gradual improvement over a period of 2 to 3 months after treatment (**C,** October 2024). At the latest review, a new thin nail plate has regrown over both big toes (**D,** April 2025).

Examination revealed moist, warty nail beds and nail matrices with anonychia and chronic paronychia of the bilateral halluces, which were better visualized on dermatoscopy (Fig 1, *A*, *B*). Pustules, vesicles, or periungual erosions were absent. The remaining skin and mucosa were uninvolved. Our initial differential diagnoses included infections such as viral wart or tuberculosis verrucosa cutis, acrodermatitis continua of Hallopeau, pyogenic granuloma, or tumor affecting both halluces, such as eccrine poroma or verrucous carcinoma. He declined a lateral longitudinal biopsy because of its perceived morbidity but agreed to 3-mm punch biopsies of the distal matrix and nail bed of the right hallux.

**Fig 2:**
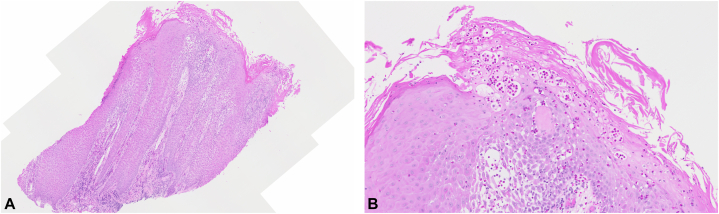
Ungual pemphigoid vegetans. A 3 mm punch biopsy of the distal nail matrix and nail bed revealed marked spongiosis and psoriasiform acanthosis with prominent intraepithelial eosinophil infiltrate, forming eosinophilic microabscesses in the subcorneal region, associated with prominent eosinophil infiltrate in the papillary dermis. (**A** and **B,** Hematoxylin-eosin stain; original magnifications: **A,** ×20; **B,** ×100.)

Histologic examination revealed marked spongiosis and psoriasiform acanthosis with prominent intraepithelial eosinophil infiltrate, forming eosinophilic microabscesses in the subcorneal region, associated with prominent eosinophil infiltrate in the papillary dermis (Fig 2, *A*, *B*). Neither acantholysis nor subepidermal blistering was seen. Tissue culture from the nail bed biopsy grew *Escherichia coli* and *Staphylococcus aureus*, whereas fungal and acid-fast bacilli tissue cultures were unyielding. The clinical presentation and histology prompted consideration of the possibility of an autoimmune blistering disease such as pemphigus vegetans of the nail unit, which could present in a similar manner.^2^, ^3^, ^4^, ^5^, ^6^ He declined a repeat biopsy of the periungual skin for direct immunofluorescence but agreed to serology testing. The serum indirect immunofluorescence revealed a roof staining pattern of 1:160 dilution on a normal human salt split skin substrate. Anti-bullous pemphigoid antigen 230 (BP230) enzyme-linked immunosorbent assay (ELISA; Euroimmun) was positive at 65.4 RU/mL (negative test if < 20 RU/mL), whereas anti-bullous pemphigoid antigen 180, anti-desmogelin 1, and anti-desmogelin 3 ELISA tests were negative.

He was treated with a 1-week course of oral cefalexin 500 mg twice daily and concurrently applied topical clobetasol propionate 0.05% cream followed by a 10% povidone iodine dressing to the affected hallux nail bed and nail matrix once daily. Gradual resolution of the paronychia and warty nail bed, and reappearance of the nail matrix and nail bed were observed after 3 months of treatment (Fig 1, *C*). Treatment was stopped when the warty lesions had completely resolved at 4 months. A new thin nail plate and reappearance of the cuticle were seen on both big toes at his latest review, 9 months after his initial presentation (Fig 1, *D*), accompanied by an improvement of his anti-BP230 ELISA titers to 50.1 RU/mL (negative test if < 20 RU/mL), whereas anti-bullous pemphigoid antigen 180 ELISA remained negative. We continue to monitor for clinical relapse of his condition with 6-monthly appointments.

## Discussion

We opined that our patient probably had isolated nail unit pemphigoid vegetans. Supportive features included the histologic finding of eosinophilic spongiosis, which is seen in early pemphigoid and pemphigus lesions; a supportive indirect immunofluorescence test; elevated anti-BP230 ELISA at 3 times the upper limit of normal; and a dramatic response to potent topical steroid therapy with a corresponding reduction of anti-BP230 ELISA titers. Our hypothesis was limited by the lack of supportive direct immunofluorescence.

Nail involvement by autoimmune blistering disease is uncommon. Paronychia, onychomadesis, Beau’s lines, onychorrhexis, onycholysis, subungual hemorrhage, and subungual hyperkeratosis are common presentations of nail unit pemphigus and, less commonly, nail unit bullous pemphigoid.^7^^,^^8^ Anonychia and pterygium formation are undesired sequelae.^7^^,^^8^ Ungual pemphigus vegetans is uncommon,^7^ whereas ungual pemphigoid vegetans is rarely reported.^9^

Pemphigoid vegetans typically presents with vegetative plaques, vesicles, or pustules in the intertriginous skin, and rarely periungual skin, with BP230 autoantibodies detected in all reported cases.^9^ Our patient presented with chronic paronychia and moist, warty plaques on the nail matrix and nail bed of both halluces. Although this has not been reported in a review of published case reports of pemphigoid vegetans,^9^ the clinical appearance of our case mirrors case reports of ungual pemphigus vegetans.^2^, ^3^, ^4^, ^5^, ^6^ This suggests a common reaction pattern of the nail unit to anti-desmoglein or anti-BP230 autoantibody-mediated eosinophilic spongiotic inflammation and onychocyte discohesion. The initial trauma on the left big toenail could have led to a loss of immune privilege of the nail matrix^10^ from exposure of matrix hemidesmosome antigens to the immune system, leading to an autoimmune response. Bacterial infection has been postulated to contribute to this vegetative presentation,^9^ similar to our case. Although BP230 autoantibody was detected, autoantibodies targeting novel non-collagenous 16a domain epitopes of bullous pemphigoid antigen 180 may also account for this clinical presentation. An immunoblot study will be useful to confirm our hypothesis.

## Conflicts of interest

None disclosed.
